# Relationship between Optical Intensity on Optical Coherence Tomography and Retinal Ischemia in Branch Retinal Vein Occlusion

**DOI:** 10.1038/s41598-018-28013-9

**Published:** 2018-06-25

**Authors:** Jian Chen, Weiqi Chen, Honghe Xia, Chuang Jin, Xuehui Lu, Haoyu Chen

**Affiliations:** Joint Shantou International Eye Center, Shantou University and the Chinese University of Hong Kong, Shantou, China

## Abstract

Branch retinal vein occlusion (BRVO) may be complicated with retinal ischemia in some cases. The purpose of the current study is to investigate the relationship between optical intensity on optical coherence tomography (OCT) and retinal ischemia in BRVO. Twenty-seven eyes diagnosed with BRVO without macular edema were classified into two groups based on the presence or absence of retinal ischemia. The optical intensity of inner retinal layers and photoreceptor inner segment ellipsoid zone/retinal pigment epithelium layer (ISe/RPE) in the affected and unaffected regions were measured on OCT. Their ratio (Optical intensity ratio, OIR) was calculated and compared between affected and unaffected region. In the retinal ischemia group, the optical intensity of inner retinal layers was higher in the affected region compared to the unaffected region while the optical intensity of ISe/RPE was low. The OIR was significantly higher in the affected region compared to control (0.83 ± 0.17 vs 0.68 ± 0.09, p < 0.001). However, in the non-ischemic group, there was no significant difference between the affected and unaffected region. The BCVA was moderately correlated with OIR of affected region (r = 0.489, p = 0.010). Our study suggests that optical intensity ratio on OCT is correlated with retinal ischemia in BRVO.

## Introduction

Retinal vein occlusion (RVO) is the second most common retinal vascular disease after diabetic retinopathy^1–4^. The Beaver Dam Study estimated the 15-year cumulative incidence of RVO at 2.3% in the population, with a majority of these (78%) being branch retinal vein occlusion (BRVO)^5^. The pathologic interruption of venous flow in BRVO patients almost occurs at retinal arteriovenous crossings, where a retinal artery crosses over a retinal vein^6–9^. About 50% to 60% of patients with BRVO are classified as ischemic type^10,11^, which is characterized by non-perfusion in the affected region. Retinal ischemia will lead to increased production of vascular endothelial growth factor (VEGF) which can cause macular edema, retinal neovascularization, tractional retinal detachment and neovascular glaucoma^12–15^.

Traditionally fundus fluorescein angiography (FFA) has been the gold standard for evaluation of retinal vasculature^16,17^. FFA is useful for identifying retinal non- perfusion and classifying ischemic vs non-ischemic BRVO. However, as an invasive technique, intravenous administration of fluorescein may result in allergic reactions such as vomiting, diarrhea, and allergic shock^18,19^. Also, it has some functional limitations, for example, it cannot show a layer by layer retinal structural change^20^.

Spectral domain Optical Coherence Tomography (SD-OCT), an *in vivo*, high-definition, medical imaging technique, is a noninvasive technology which provides cross-sectional images of the retinal microstructure^21,22^. Several studies have reported that retinal ischemia associated with diabetic retinopathy and RVO results in thinning of the inner retina^23–25^. An increase in the optical intensity in inner retinal layers is observed qualitatively in retinal ischemic disorders, such as retinal artery occlusion^26,27^, diabetic retinopathy, retinal vein occlusion, hypertensive retinopathy, radiation retinopathy, HIV retinopathy^28^, Purtscher’s retinopathy, and Purtscher like retinopathy^29,30^. In our previous study^26,27^, we quantitatively analyzed the optical intensity of retinal layers in central retinal artery occlusion (CRAO). The results showed that optical intensity at inner retinal layers increases, including retinal ganglion cell layer, inner plexiform layer, and inner nuclear layer/outer plexiform layer. The optical intensity at photoreceptor inner segment ellipsoid zone and retinal pigment epithelium (ISe/RPE) layer decreased. The ratio between the two optical intensity values was highly correlated with visual prognosis.

Retinal ischemia in BRVO may share similar characteristics with CRAO. The purpose of the current study is to investigate the relationship between the optical intensity on OCT with retinal ischemia and visual acuity in BRVO.

## Results

Twenty-seven eyes from 27 patients (14 males and 13 females) were included in this study. The mean age was 60.15 ± 9.23 years (range: 44–81 years). The demographic information, clinical and optical coherence tomographic characteristics are shown in Table 1. Table 2 showed the comparison of characteristics between the eyes with and without retinal ischemia. The mean age in ischemic group was significantly younger compared to the non-ischemic group (57.0 ± 9.5 vs 66.1 ± 7.0, p = 0.009). The BCVA was slightly worse in the ischemic group compared to the non-ischemic group, however the difference was not statistically significant.

**Table 1:** Characters of Included Subjects.

Group	NO.	Gender	Age (year)	Eye	Optical intensity						BCVA (LogMAR)
					Inner retina		ISe/RPE		Ratio		
					affected	unaffected	affected	unaffected	affected	unaffected	
Non-ischemia	1	M	53	OS	78.69	101.81	150.91	173.30	0.52	0.59	0.1
Non-ischemia	2	M	81	OD	117.10	115.32	155.77	172.86	0.75	0.67	0.4
Non-ischemia	3	F	61	OS	70.60	62.25	116.97	101.66	0.60	0.61	0.2
Non-ischemia	4	M	67	OS	89.08	90.09	139.91	142.98	0.64	0.63	0.3
Non-ischemia	5	M	67	OD	97.31	99.09	141.72	142.21	0.69	0.70	1.1
Non-ischemia	6	F	70	OD	96.57	80.85	120.83	115.67	0.80	0.70	0.3
Non-ischemia	7	F	66	OD	107.47	87.31	159.20	139.54	0.68	0.63	0.2
Non-ischemia	8	M	67	OD	98.10	87.44	105.42	116.73	0.93	0.75	0.7
Non-ischemia	9	M	65	OD	51.90	99.75	123.19	134.17	0.42	0.74	0.2
Non-ischemia	10	M	64	OD	84.00	76.67	125.89	131.57	0.67	0.58	0.3
Ischemia	11	M	63	OS	83.28	85.27	105.77	103.83	0.79	0.82	1.1
Ischemia	12	F	49	OS	112.73	86.16	114.36	106.58	0.99	0.81	0.6
Ischemia	13	M	66	OS	102.68	110.76	104.75	165.44	0.98	0.67	0.4
Ischemia	14	F	44	OS	116.84	100.33	127.34	158.17	0.92	0.63	0.2
Ischemia	15	F	64	OD	124.29	114.13	156.47	161.64	0.79	0.71	0.6
Ischemia	16	M	49	OD	140.01	110.23	137.98	169.14	1.01	0.65	0.3
Ischemia	17	F	49	OD	102.04	89.24	156.32	155.29	0.65	0.57	0.3
Ischemia	18	F	55	OS	132.06	133.00	139.56	181.61	0.95	0.73	0.8
Ischemia	19	F	54	OS	119.87	116.94	147.84	157.83	0.81	0.74	0.1
Ischemia	20	M	64	OS	94.98	106.05	133.77	165.59	0.71	0.64	0.1
Ischemia	21	F	49	OD	95.40	84.16	147.39	121.30	0.65	0.69	0.1
Ischemia	22	F	68	OS	99.68	84.58	128.46	137.29	0.78	0.62	0.1
Ischemia	23	M	79	OS	94.02	79.25	151.99	144.10	0.62	0.55	0.7
Ischemia	24	M	63	OS	161.04	132.98	146.23	174.22	1.10	0.76	1.4
Ischemia	25	F	49	OD	105.44	110.03	99.41	148.45	1.06	0.74	0.7
Ischemia	26	M	48	OS	96.00	102.40	116.73	154.16	0.82	0.66	0.8
Ischemia	27	F	56	OS	72.72	57.16	144.69	122.11	0.50	0.47	0.2

Inner layers: including retinal nerve fiber layer, ganglion cell layer, inner plexiform layer, inner nuclear layer, outer plexiform layer; ISe/RPE: photoreceptor inner segment ellipsoid zone/retinal pigment epithelium; BCVA: best-corrected visual acuity; M: male; F: female.

**Table 2:** Comparison of the characters between BRVO with and without retinal ischemia.

	Ischemia	Non-ischemia	p	Statistics
N	17	10		
Gender (M: F)	7:10	7:3	0.148	Chi-square
Age (year)	57.0 ± 9.5	66.1 ± 7.0	0.014	t test
Inner retina affected region	109.0 ± 21.8	89.1 ± 18.8	0.024	t test
Inner retina unaffected region	100.2 ± 19.9	90.1 ± 14.9	0.177	t test
ISe/RPE disease part	132.9 ± 18.7	134.0 ± 18.1	0.883	t test
ISe/RPE control part	148.6 ± 23.1	137.1 ± 23.2	0.221	t test
OIR disease part	0.83 ± 0.17	0.67 ± 0.14	0.018	t test
OIR control part	0.68 ± 0.09	0.66 ± 0.06	0.636	t test
BCVA (LogMAR)	0.50 ± 0.38	0.38 ± 0.30	0.604	U test

BRVO: branch retinal vein occlusion; ISe/RPE: photoreceptor inner segment ellipsoid zone/retinal pigment epithelium; BCVA: best-corrected visual acuity; M: male; F: female.

The ICC of optical intensity measurement was high in all regions of interest (ICC = 0.986, 0.994, 0.908, 0.968 for the inner retina at affected region, ISe/RPE at affected region, inner retina at unaffected region and IS/RPE at unaffected region respectively).

**Figure 1:**
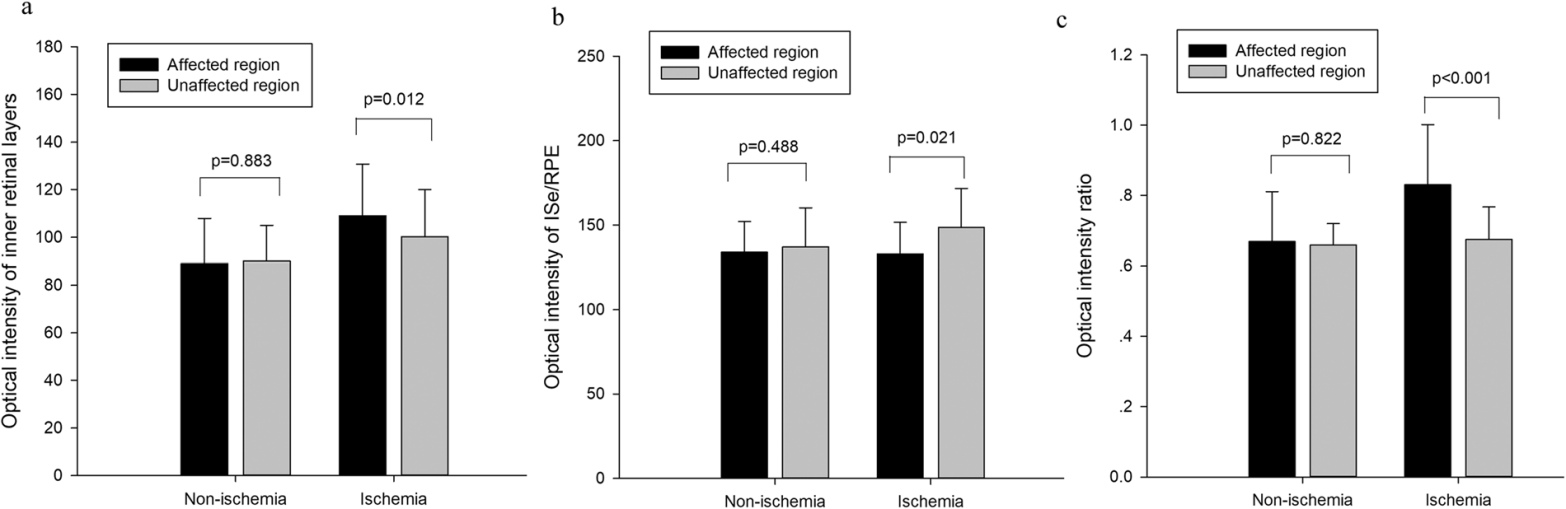
Comparisons of optical intensities and their ratio between affected and unaffected regions in BRVO eyes in the groups of retinal ischemia and non-ischemia. (**a**) optical intensity of inner retinal layers. (**b**) optical intensity of Inner segment ellipsoid zone (ISe) and retinal pigment epithelium (RPE). (**c**) optical intensity ratio of inner retina vs. ISe/RPE. The error bars represent standard deviation. The p values were calculated using paired t test.

**Figure 2:**
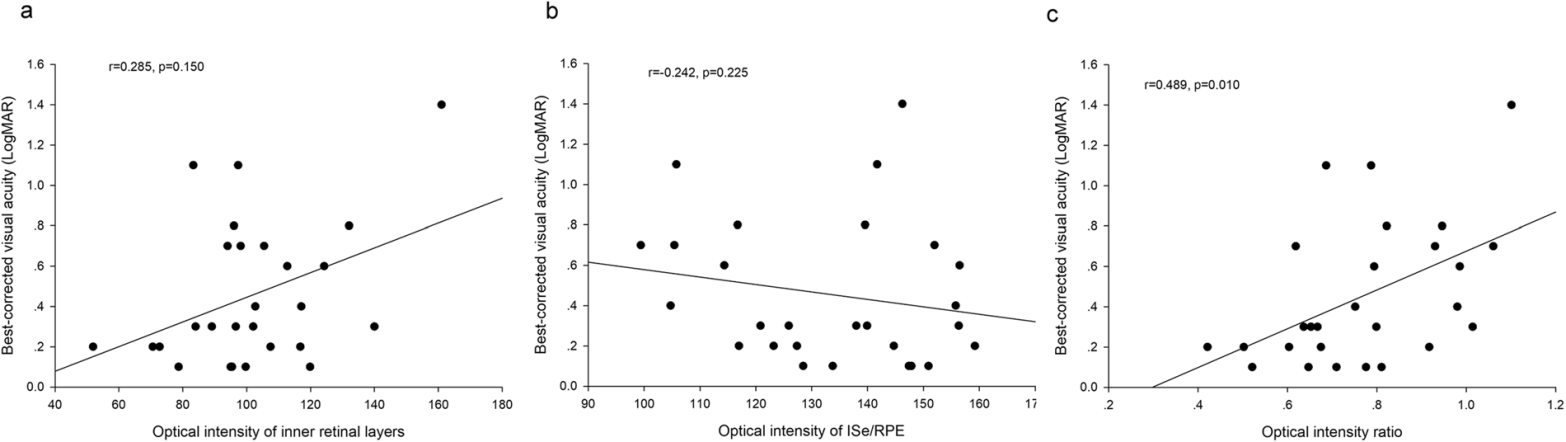
Scatter plots of the correlation between best-corrected visual acuity and (**a**) optical intensity of inner retinal layers, (**b**) optical intensity of Inner segment ellipsoid zone (ISe) and retinal pigment epithelium (RPE), (**c**) optical intensity ratio of inner retina vs ISe/RPE.

The comparison of OCT characteristics between affected and unaffected region is shown in Fig. 1. In the non-ischemic group, there was no significant difference between optical intensity of affected region and that of unaffected region in inner retina or ISe/RPE, or their ratio (all the p > 0.05). In the ischemic group, the optical intensity of affected region was significantly higher than that of unaffected region in inner retina (109.0 ± 21.8 vs 100.1 ± 20.0, p = 0.012), and lower in ISe/RPE (132.9 ± 18.7 vs 148.6 ± 23.1, p = 0.021). The OIR was significantly higher in the affected region compared to that of unaffected region (0.83 ± 0.17 vs 0.68 ± 0.09, p < 0.001). Among all the BRVO patients, the OIR of affected region was positively correlated with BCVA (r = 0.489, p = 0.010, Fig. 2).

## Discussion

Our study found that in regions with retinal ischemia in BRVO, the optical intensity of inner retinal layers increased, the intensity in outer retina decreased and the OIR increased. Furthermore, OIR was moderately correlated with BCVA.

SD-OCT has been widely used in evaluation of retinal diseases including BRVO, however, most of the published studies focused on retinal thickness. The cross-sectional images on OCT can demonstrate intraretinal cysts, exudative retinal detachment, and retinal thickening^31^. OCT can also provide quantitative measurement of retinal thickness with is useful for quantifying the disease severity and follow up of patients with and without treatment^32^. Change in retinal optical intensity or reflectivity has also been reported^33^ but not quantitatively investigated. This study quantified the optical intensity of retinal inner and outer layers in the diseased and unaffected region. Our results showed that in ischemic BRVO, the optical intensity increased in the inner retinal layers and decreased in the outer retina, while there was no difference of optical intensity in non-ischemic BRVO.

OCT angiography has been recently reported in investigation of retinal ischemia in BRVO^34,35^. It needs special modules and is not commonly available in clinical practice unlike SD-OCT which is widely used in clinics. Furthermore, FFA and OCT angiography only outline the range of retinal non-perfusion. The information demonstrated on OCTA represents flow signal, while the change of intensity represents the tissue response to ischemia. Our study found that the OIR was correlated with visual acuity, which suggests that OIR may represent the severity of retinal ischemia in BRVO.

Change in optical intensity was not only seen in BRVO, but also in other retinal ischemic diseases. Our previous studies found similar results in central retinal artery occlusion^26,27^. The exact mechanism of optical intensity change in retinal ischemia remains unknown. It was suggested that the increased optical intensity in inner retinal layers may represent glial transformation^28^. The change in intensity is noted across the regions of retinal ischemia and is localized in inner retinal layers only corresponding to the retinal vasculature. It was also suggested that this change is due to intracellular edema caused by retinal ischemia^36^. The reduced intensity of outer retina may be due to the shadowing effect caused by increased optical intensity in the inner retina^26^

Although these OCT findings have potential use for noninvasive evaluation of macular ischemia, the current study had a few limitations. First, the sample size was small and further study with larger sample size is needed to confirm our results. Second, our study included only BRVO patients without macular edema. Further studies are needed to investigate the change of optical intensity in BRVO with macular edema. Third, our study is a cross-sectional study. Further studies are needed to investigate the longitudinal change of optical intensity and its functional correlation. Fourth, visual acuity was measured using decimal chart and converted to LogMAR. This may affect the accuracy of visual acuity measurement. Fifth, our study used manual segmentation for 8 bit 2D image. Further studies are needed to investigate the optical intensity using automatic segmentation for 16 bit 3D data. Fifth, two OCT systems were used in this study. They may have different hardware and algorithm, which may affect the result of optical intensity. However, optical intensity ratio is a normalized parameter with internal reference. A study compared retinal layer intensity profiles from different OCT machines found that following normalization, the differences between OCT devices were no longer significant^37^. Sixth, the shadowing effect of blood vessel may affect the results. Although the blood vessels are roughly symmetrical on the superior and inferior part of retina on the vertical OCT scan across the fovea, the effect of blood vessel is not exactly the same on both sides.

In conclusion, the present study identified that optical intensity changes in retinal ischemia in patients with BRVO patients without macular edema. We reported that OIR on OCT can be used as a noninvasive evaluation of macular ischemia in BRVO.

## Methods

The study adhered to the tenets of Declaration of Helsinki, and was approved by the Institutional Review Board of Joint Shantou International Eye Center of Shantou University and the Chinese University of Hong Kong. Because of its retrospective nature, informed consent was waived.

### Study Subjects

The patient database in Joint Shantou International Eye Center was searched and patient records between January 2012 and August 2016 were reviewed. Patients included were those who met the following criteria: (1) Super-temporal or infer-temporal BRVO with macula involved; (2) The interval between FFA and OCT was less than 1 month; (3) To exclude the effect of macular edema on the measurement of optical intensity and visual function, we included cases without macular edema. The exclusion criteria were (1) OCT image quality score <4/10 in ZEISS or <40/100 in TOPCON; (2) since retinal hemorrhage may affect optical intensity, eyes with retinal hemorrhage within the scanned region were excluded; (3) Presence of age-related macular degeneration, diabetic retinopathy, retinal artery occlusion, or myopic retinopathy; (4) History of ocular trauma.

**Figure 3:**
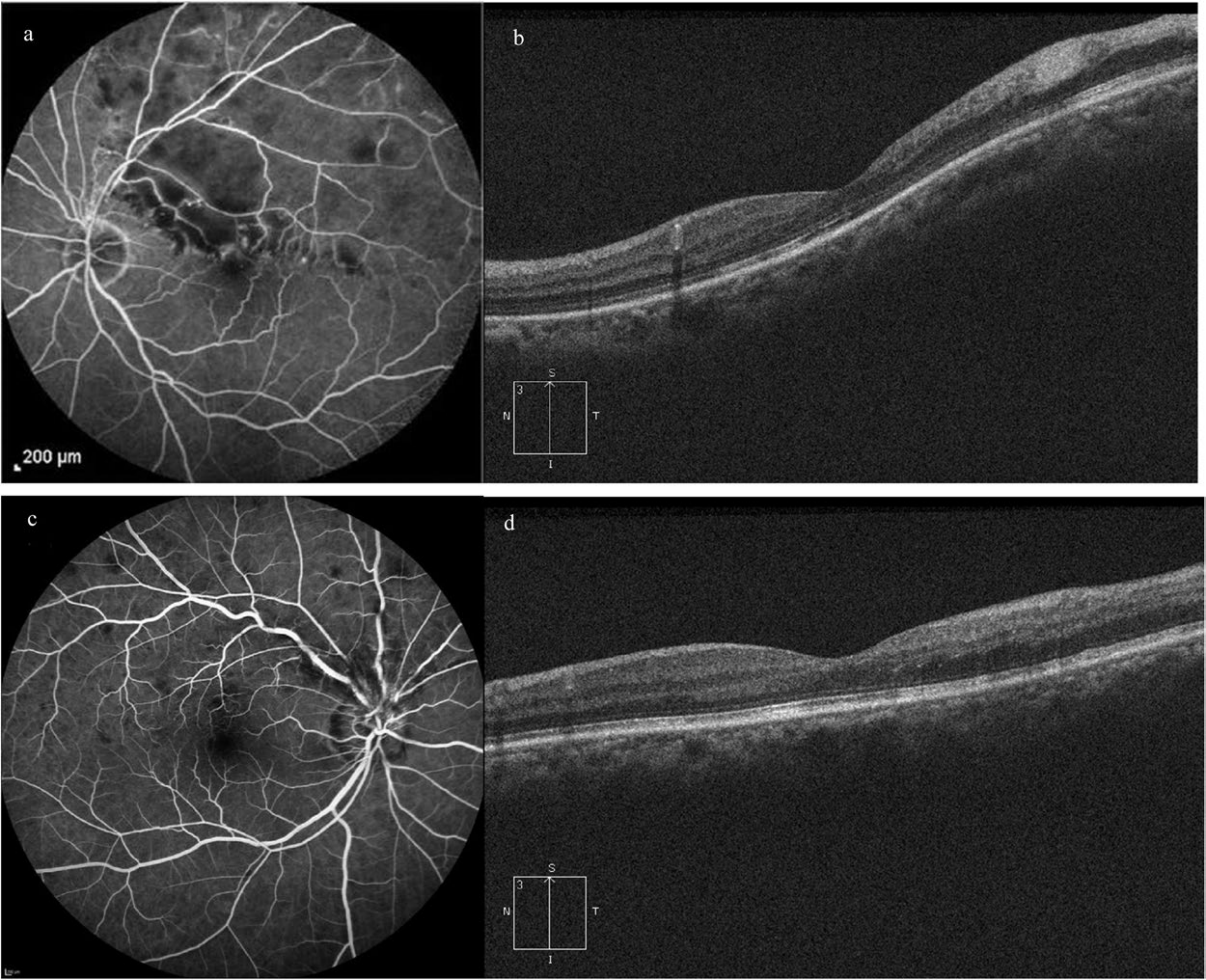
Fundus Fluorescein Angiography venous phase images (**a**,**c**) and corresponding SD-OCT vertical scan images (**b**,**d**) of two patients with branch retinal vein occlusion. (**a**) Retinal non-perfusion can be identified at supratemporal region. (**b**) Increased optical intensity can be identified at inner retina and reduced optical intensity at ellipsoid zone and retinal pigment epithelium. (**c**) There is no retinal nonperfusion. (**d**) no obvious change of optical intensity can be seen.

All patients included in this study underwent comprehensive ophthalmic examinations, including best-corrected visual acuity (BCVA), noncontact tonometry, slit-lamp biomicroscope, fundus photography, FFA and SD-OCT examination. The patients were then classified into two groups based on the presence or absence of retinal non-perfusion on FFA (Fig. 3).

### OCT Examinations

SD-OCT examination was performed using two OCT machines. (1) Topcon 3D OCT-1000 (Topcon Corporation, Tokyo, Japan). Macula was scanned using 6 mm radial scan mode, and the vertical scan across fovea was chosen. (2) ZEISS Cirrus OCT (Carl Zeiss Meditec, Dublin, California, United States). The macular region was scanned using the vertical HD 5 Line Raster scan (6 mm) mode and the scan across fovea was chosen. The OCT images were exported as grayscale images from the OCT software (BMP from ZEISS, JPEG from Topcon). OCT image quality score was provided by the on-board OCT software.

**Figure 4:**
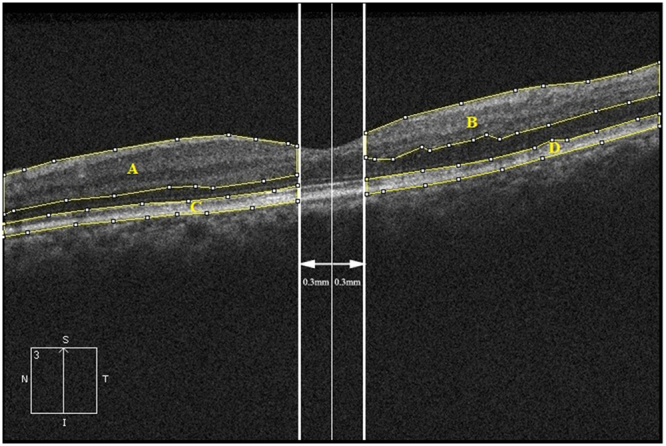
Definition of regions of interest for measurement of the optical intensity on spectral-domain optical coherence tomography image of branch retinal vein occlusion. (**A**,**B**) Inner retinal layers, including the retinal nerve fiber layer, ganglion cell layer, inner plexiform layer, inner nuclear layer, and outer plexiform layer; (**C**,**D**) Inner segment ellipsoid zone and retinal pigment epithelium. (**A**,**C**) unaffected region. (**B**,**D**) affected region.

The optical intensity measurement was obtained using Image J software (National Institute of Health, Bethesda, MD)^38^ using previously described methods^39,40^. Regions of interest were manually selected by two independent masked investigators. The images were divided into affected region and unaffected region by the vertical line passing through the fovea. The foveal avascular zone (with diameter of 0.6 mm) was excluded from the analysis because there is no retinal inner layer and there may be disruption of ISe which would affect the measurement of optical intensity and produce cofounding effect on BCVA. In each part, we defined two regions of interest, inner retinal layers and ISe/RPE (Fig. 4). The optical intensity of each ROI was measured using Image J. The optical intensity is the gray level of selected regions on a scale of 0 (pure black) to 255 (pure white). The ratio of optical intensity of inner retinal layers and the ISe/RPE was calculated as OIR.

### Statistical Analysis

Data for continuous variables were expressed as mean ± standard deviation. Visual acuity measurements were converted to logarithm of the minimal angle of resolution (LogMAR) for all analyses using the following formula: LogMAR = −log (Snellen decimal acuity). The reliability of optical intensity of each regions of interest was assessed by calculating the intraclass correlation (ICC) of the two readers. And their means were used for further analysis.

The distribution of continuous variables was assessed using Shapiro-Wilk test. The demographic and clinical characters were compared between the groups with and without retinal ischemia using independent t test, except BCVA, which was compared using Mann-Whitney U test. Within each group, the optical intensity of inner retinal layers, ISe/RPE, and optical intensity ratio were compared between the affected and the unaffected region of the same eye using paired t test. Correlations between the OIR at affected region with BCVA were assessed by Spearman correlation. P value less than 0.05 is considered as statistically significant. Statistical analyses were performed using SPSS version 16.0 for Windows (SPSS, Inc, Chicago, IL).

### Summery

The optical intensity on OCT increased in the inner layers and decreased in the outer layers in ischemic BRVO and their ratio was correlated with BCVA. The optical intensity did not change in non-ischemic BRVO.
